# Supplementary material for: Retention of zirconia crown to oxidized titanium base abutment: Experimental research
Source: PLoS One. 2023 Oct 25;18(10):e0287108. doi: 10.1371/journal.pone.0287108 (PMC10599539; doi:10.1371/journal.pone.0287108)
Supplement: Attachment — Submitted filename: Response to reviewer.docx [file pone.0287108.s004.docx]

To the Editor and reviewers of the PLOS ONE journal

Dear Editor and reviewers,

Thank you for taking the time to review my manuscript. I appreciate your feedback; they were accommodating. I have incorporated all of your suggestions in my revisitation as described in this letter.

1) Please add SEM method in the Methods section.

Answer: I have add SEM and EDS method in the Method section(page5 line 141-146)

2) Since you compared the SEM analyses among different groups, I think we need to take it one step further to see if there is damages or changes in material elemental composition. Can you add EDX experiments into this? If not please provide justification and rationale. My thought is that air abrasion may alter the composition of the abutment surface.

Answer: I have add the EDS analysis and add the result graph demonstrate material composition along with a table demonstrate element composition in weight% and atomic%.

I also discuss finding from EDS on increasing of aluminum and oxygen after air abrasion. As reference “23. Ohkubo C, Watanabe I, Hosoi T, Okabe T. Shear bond strengths of polymethyl methacrylate to cast titanium and cobalt-chromium frameworks using five metal primers. The Journal of prosthetic dentistry. 2000 Jan 1;83(1):50-7.” After air abrasion, Al_2_O_3_ particles embedded into metal surface which increase resin bond strength.

3) Please change "sand blasting" to "air abrasion".

Answer: I have change all “sandblasting” to “air abrasion” including the name of groups “sandblasted group” to “air abraded group” and “Oxidized-sandblasted group” to “oxidized-air abraded group” for consistency of the manuscript.

4) Can you discuss the depth of the air abrasion and the anodization layer? I assume that air abrasion would not go beyond the anodization layer. If we will recommend this clinically, would this be an issue if someone air abrade the implant abutment too much?

Answer: Since this study did not investigate on the anodized abutment, we couldn’t make any assumption of this matter. Further research should be conducted to investigate the effect of air-abraded on anodized abutment.

Finally, I appreciate all your feedbacks. Thank you for your time and consideration.

Sincerely,

Assistant Professor Chatchai Kunavisarut

Business address: Department of Advanced General Dentistry, Mahidol University,

No.6 Yothi Road, Ratchathewi District, Bangkok 10400 Hospital (Phayathai Campas)

Tel:+662-200-7777

Email: [Chatchai.kun@mahidol.ac.th](mailto:Chatchai.kun@mahidol.ac.th) Mobile:+66890737030
